# CSF1R defines the mononuclear phagocyte system lineage in human blood in health and COVID-19

**DOI:** 10.1093/immadv/ltab003

**Published:** 2021-02-17

**Authors:** Theo W Combes, Federica Orsenigo, Alexander Stewart, A S Jeewaka R Mendis, Deborah Dunn-Walters, Siamon Gordon, Fernando O Martinez

**Affiliations:** 1 Faculty of Health and Medical Sciences, University of Surrey, Guildford, UK; 2 Department of Biotechnology and Biosciences, Università degli Studi di Milano-Bicocca, Milan, Italy; 3 Clinical Trials Unit, University of Surrey, Guildford, UK; 4 Graduate Institute of Biomedical Sciences, College of Medicine, Chang Gung University, Taoyuan City, Taiwan; 5 Sir William Dunn School of Pathology, University of Oxford, Oxford, UK

**Keywords:** CSF1R, monocyte, dendritic cells, COVID-19, blood, human

## Abstract

Mononuclear phagocytes defend tissues, present antigens, and mediate recovery and healing. To date, we lack a marker to unify mononuclear phagocytes in humans or that informs us about their origin. Here, we reassess mononuclear phagocyte ontogeny in human blood through the lineage receptor CSF1R, in the steady state and in COVID-19. We define CSF1R as the first sensitive and reproducible pan-phagocyte lineage marker, to identify and enumerate all conventional monocytes, and the myeloid dendritic cells. In the steady state, CSF1R is sufficient for sorting and immuno-magnetic isolation. In pathology, changes in CSF1R are more sensitive than CD14 and CD16. In COVID-19, a significant drop in membrane CSF1R is useful for stratifying patients, beyond the power of cell categories published thus far, which fail to capture COVID-19 specific events. Importantly, CSF1R defines cells which are neither conventional monocytes nor DCs, which are missed in published analysis. CSF1R decrease can be linked *ex vivo* to high CSF1 levels. Blood assessment of CSF1R+ cells opens a developmental window to the Mononuclear Phagocyte System in transit from bone marrow to tissues, supports isolation and phenotypic characterisation, identifies novel cell types, and singles out CSF1R inhibition as therapeutic target in COVID-19 and other diseases.

## Graphical Abstract

**Graphical Abstract:**
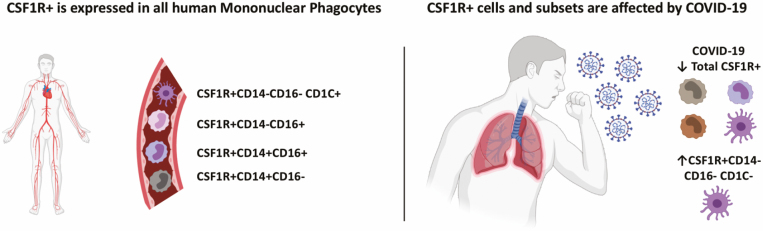

## Introduction

The mononuclear phagocyte system (MPS) is a dispersed homeostatic organ of embryonic and bone marrow origin [1]. Blood monocytes present an accessible window on the MPS, providing information about their haematopoietic origin, as they transit to tissues to become heterogeneous effector macrophages. With updated analytic tools, we have reassessed the expression of lineage determining cytokine receptors (LDCRs) in human blood in the steady state and in disease.

Mononuclear phagocytes are the only human cellular system for which we lack a unified marker and origin theory [2, 3]. CD14, CD16, and CD1c are terminal markers which describe monocyte subsets and dendritic cells (DCs), but keep them apart as a single cell family. Through the years, CD14 CD16 subset characterisation has been done without full understanding of these heterogeneous cells. A consensus is beginning to emerge for CD14 and CD16, their extremes and intermediate subsets, but this is limited in scope since there is mounting evidence for more than three populations of monocytes [2, 4–6]. Independently, the field of DCs has exploded into several families, despite representing less than 3% of the family in the steady state [4]. Here, the biggest challenge is finding a unifying marker to distinguish them from the rest of white blood cells.

Current manuscripts use overcomplicated gating strategies to capture the MPS cells, and do not go beyond established markers [7]. Strategies in place include numerous markers, including CD88 and CD89 which are actually higher in polymorphonuclear cells than monocytes and HLA molecules which are expressed by all members of the family at protein level [4], including monocytes, and the controversial CD11c [8). Further obscuring facts are that selected DCs express CD14, CD16, CD163, and not all express CD1c, making it impossible to capture or subset the cell family in respect to monocytes, in a consistent manner. In COVID-19 research, the same issue arises and many of the reports simply apply what is known about existing markers, failing to integrate parameters with logistic statistical models, therefore losing important information about novel cell types and more informative marker combinations for the disease.

Monocytes constitute an important innate cell network that provides tissues as needed, with inflammatory, pro-resolution macrophages and myeloid-derived suppressor cells. Lineage markers, e.g. CD3 as pan T cell marker, CD19 and CD20 for B cells and CD56 for NK cells, exist for all but the cells of the mononuclear phagocyte system. With updated methods and analytic tools, we reassign CSF1R as the first reproducible pan-mononuclear phagocyte system marker in humans. Furthermore, we clarify the cellular targets of the drugs that are being developed for CSF1R, with profound clinical implications. In COVID-19, the CSF1R system is the most informative MPS marker and makes it possible to distinguish patients from other pulmonary diseases. Dissecting the contribution of CSF1R expression to human blood and tissues, will enable us to develop anti-COVID-19, anti-cancer, and anti-inflammatory strategies, rescuing the MPS for therapy.

## Materials and Methods

### Blood cell preparation

Peripheral venous blood was taken with informed consent from normal volunteers in accordance with ethical approval UEC/2017/052/FHMS at the University of Surrey. Blood was processed within 2 hours of venepuncture. Blood was collected in vacutainers containing sodium heparin (BD Biosciences). PBMCs were isolated from whole blood by standard density centrifugation using Ficoll-Paque (GE-Healthcare).

### COVID-19 cohort

Participants included in this study were recruited at NHS Frimley Park Hospital (Frimley, Surrey, England). Collection of the blood was performed by researchers from University of Surrey at NHS Frimley Park Hospital between May and September 2020. Ethical approval for this project (IRAS project ID 155921) was obtained via the NHS Health Research Authority (REC reference: 14/LO/1221). Samples were transferred from the Hospital to the University of Surrey by courier at controlled temperature (4°C) and processed within 4 hours of collection. Alongside blood collection, metadata for all participants was also collected covering inter alia gender, age, comorbidities (based on whether the participant was receiving treatment), the results and dates of COVID PCR (polymerase chain reaction) tests, bilateral chest X-ray changes, smoking status, and whether the participant presented with clinical symptoms of COVID. A total of 71 patients and 10 healthy controls took part to this study. Demographics (Supplementary Table S1) is presented in the Supplementary Material.

### Flow cytometry and cell sorting

Leukocytes were stained in fresh whole blood, in 100 μl aliquots, with the following antibodies (all from Biolegend unless otherwise stated): BV421-anti-CSF1R; BV510-anti-CD16; BV650-anti-CD14; BV786-anti-IL3RA; FITC-anti-CSF2R; PE-anti-IL15RA; PE-Dazzle-anti-IL7R; PerCP/Cy5.5-anti-FLT3; APC-anti-CSF3R; FITC-anti-CD56, FITC- anti-CD19 (eBioscience); APC-anti-CD3, FITC-anti-CD1c, AF700-anti-HLA-DR. All antibodies were added at 1 in 100 dilution and cells incubated for 20 minutes at RT. Subsequently, erythrocytes were lysed, and leukocytes fixed with RBC fix/lysis buffer (Biolegend). Cells were washed with PBS, without Ca^2+^ and Mg^2+^, and read on a triple-laser BD FACS Celesta (BD Bioscience). PBMC staining was performed in a similar fashion, with the addition of a blocking step with Human TruStain FcX™ (Biolegend).

Cell populations were distinguished using forward light scatter (FSC-A) versus side scatter (SSC-A), and then gated for single cells using forward light scatter height (FSC-H) versus area, and live cells using the live-dead stain Zombie NIR (Biolegend), as per manufacturer’s instructions. Sorter tuning and power were kept consistent across all measurements. Panel compensation was done with single stained cells. Flow cytometry analysis was performed using Flowjo^TM^. Cell sorting was performed on a four laser BD FACS Aria (BD Bioscience). Cell populations were gated using CSF1R, followed by gating by CD14 and CD16. Representative plots of single stained cells can be found in Supplementary Fig. 1. Isotype-matched controls and Fluorescence minus one (FMO) samples were used. This protocol is optimised for working on fresh blood and PBMCs.

### Monocyte stimulations

Short-term stimulation assays were performed in 100 µl whole blood with 100 ng/ml CSF1 (Biolegend) and 100 ng/ml LPS *Escherichia coli* K12 (Invivogen). Longer stimulation assays (48 hours and 7 days) were performed on PBMCs in polypropylene plates in RPMI-1640 media supplemented with 10% FCS (Sigma). The 48 hours stimulation utilised the following immune stimuli: 100 ng/ml CSF1, 100 ng/ml IL-4, 100 ng/ml IFNγ (all Biolegend), 500 nM Dexamethasone (Sigma), 100 ng/ml LPS, 100 ng/ml PAM3CSK, 100 ng/ml FLAG, 100 ng/ml FSL1, and 10^8^ Listeria cells (All Invitrogen). After stimulation, cells were harvested, and stained as above. The 7-day stimulation utilised the following LDCs: CSF1, IL-34, CSF2, CSF3, IL-3, IL-7, IL-15, and FLT3L (All 25 ng/ml, Biolegend). After stimulation, cells were harvested and stained as above.

### Single cell RNAseq analysis

PBMCs were sorted on the BD FACS Aria. We selected CSF1R+ cells and separated the cells based on CD14 and CD16 expression. We isolated cells from each quadrant including the CD14-CD16- quadrant. Cells from each quadrant were equalised, this enriched the rare double negative, intermediate and CD16 population of monocytes. Subsequent single cell emulsions and barcoding workflow were performed using 10× genomics 5’ capture kit according to user guide CG000086. Samples were sequenced on a HiSeq2500 High Output in the 30-10-100 format at one sample per lane. CellRanger3.1.0 was used to annotate the transcriptome generating: 6153 cells, median 2141 genes per cell, and 6490 Unique Molecular Identifier (UMI) counts per cell. Single-cell sequencing data were analysed with the Seurat [42] R package. Briefly, 3,714 cells were selected that had: >500 unique RNA features, <10% mitochondrial genes, lacked MS4A1, CD3E, GNLY, PPBP, CD8A. The samples were normalised using scTransform and percent mitochondrial genes regressed out, the Harmony package was then used to regress-out patient variation. PCA and UMAP (umap-learn, correlation, 15 dims, others as default) were used for multidimensional analysis. For FindNeighbour and FindCluster functions, the default settings were used. A hierarchical tree matrix was constructed using the BuildClusterTree function in Seurat using PCA at the level of the nearest neighbour clusters; UMAP cluster IDs were then releveled according to this phylogeny. Plots and differential gene expression (min.pct = 0.25, as standard) were calculated in *Seurat*. Data are deposited in geo profiles link GSE161738.

### Statistics

Statistical analyses were performed on GraphPad PRISM 8 programme. Specific tests for each comparison are indicated in the corresponding figure legends. Graphs were plotted consistently using the mean bars and standard deviation as error. Logistic analysis was performed in SAS.

## Results and Discussion

**Figure 1:**
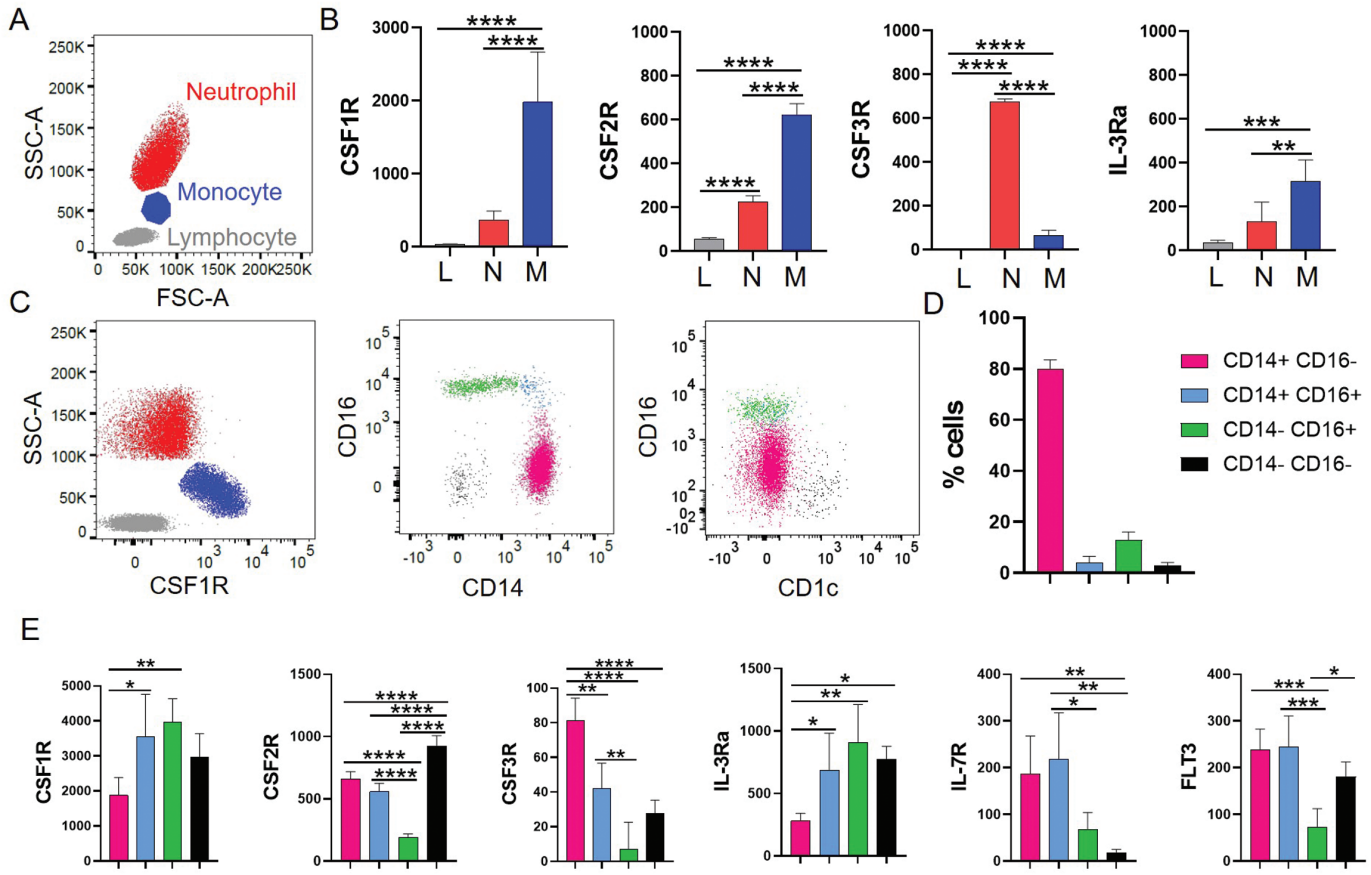
CSF1R is a pan phagocyte marker in human blood. Whole blood was stained with a panel of antibodies targeting LDCRs. (A) Representative SSC-A versus FSC-A dot plot displaying the three main leukocytes found in whole blood: lymphocytes in grey, monocytes in blue, and neutrophils in red. (B) We show the median fluorescent intensity of four LDCRs CSF1R, CSF2R, CSF3R, and IL-3Ra in the leukocytes. (C) Representative dot plot of CSF1R expression in whole blood. Next is a representative dot plot of CSF1R+ cells separated with CD14 and CD16. A fourth population of CSF1R+ CD14- CD16- population can be identified, shown in black. Representative dot plot of CSF1R+ cells separated with CD16 and the DC marker CD1c+, highlights that the CSF1R+CD14- CD16- cells are CD1c+. (D) The distribution (%) of CSF1R+ cells into CD14 CD16 is shown. Bar colours match the populations in the dot plot. All bar charts in the figure represent the mean with SD (*n* = 5). (E) Expression of the six LDCRs in the subsets. We plotted the MFI for each receptor with SD (*n* = 5). Statistics were assessed with one-way ANOVA and Tukey multiple comparison test; *P < 0.05; **P < 0.0021; ***P < 0.0002; ****P < 0.0001.

To establish an MPS classification which more closely reflects their origin, we focused on establishing and measuring the LDCRs which drive monocyte development in human bone marrow [9], and are expressed in the periphery. We identified seven receptors, myeloid-specific CSF1R [10–13], CSF2R [14], CSF3R [15], and more general, FLT3 [16, 17], interleukin 3 receptor (IL3R) [18, 19], IL15R [20, 21], and interleukin 7 receptor (IL7R) [22–24]. By whole blood fluorescent activated cell sorting (FACS), CSF1R displays highest expression in monocytes [median fluorescent intensity (MFI): 1983], distinct from lymphocytes and neutrophils (MFI: 366), Fig. 1A and B and Supplementary Fig. S1. CSF2R was also expressed significantly higher in monocytes (MFI: 623) versus neutrophils (MFI: 227), and lymphocytes. CSF3R was expressed at significantly higher levels in neutrophils (MFI: 675) than monocytes (MFI: 68). Cohen’s *d* calculation ranking showed CSF1R, CSF2R, CSF3R, and IL-3Ra as monocyte markers with the greatest signal over noise.

Mononuclear phagocytes are commonly defined by CD14 and CD16 for monocytes and CD1c for the most abundant myeloid DCs. CSF1R captures all three conventional monocytes, CD14+CD16-, CD14+ CD16+, and CD14-CD16+, and additionally, a CD14-CD16-CD1c+ population, which represents circa 3% of total monocytes in the steady state, Fig. 1C and D. CSF2R in comparison captured mostly CD14+ cells (Supplementary Fig. S2).

We then investigated LDCR expression in each subset (Fig. 1E). CSF1R was expressed at high levels on all subsets, but was highest on CD16+ cells (CD14-CD16+ and CD14+CD16+ ~MFI: 3700) and DCs. IL3R had similar expression to CSF1R, with high expression in the CD16+ monocytes and DCs (~MFI: 700). Both cytokines are used *in vivo* and *in vitro* to generate monocytes [25]. Despite these similarities, the most distinctive population in LDCR terms is the CD14- CD16+ monocyte; this cell has lower levels of CSF2R, CSF3R, IL7R, and FLT3, compared with the remaining subsets. The CD14-CD16- DCs have the highest levels of CSF2R of all subsets (MFI: 920), but lack IL7R. CSF2 is used to generate DCs and IL-7R lack can be useful to distinguish bona fide DCs versus monocyte-derived DCs [26]. In general, CD14+ monocytes stained high for CSF2R (MFI: 654) and CSF3R (MFI:75). CSF3R expression in CD14+ monocytes has been linked with a neutrophil-like phenotype [27].

**Figure 2:**
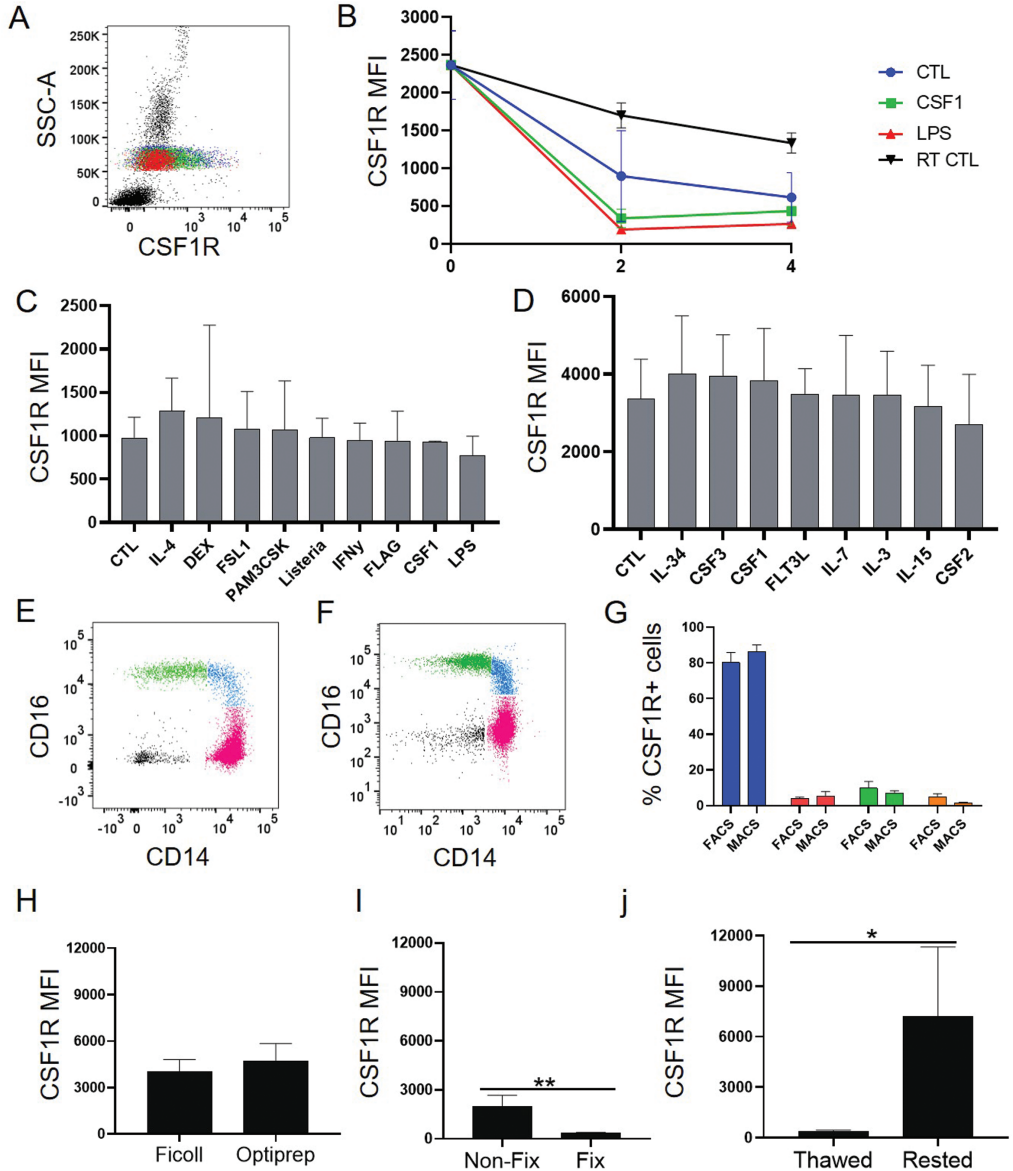
CSF1R is a robust yet sensitive marker in human blood. (A) FACS plot of blood exposed to CSF1, LPS, or left at room temperature for 2 hours. (B) CSF1R expression quantification in whole blood samples stimulated with CSF1 or LPS for 2 and 4 hours at 37°C. (C) CSF1R expression in monocytes stimulated for 48 hours with several immune stimuli. (D) CSF1R expression in monocytes stimulated for 7 days with lineage determining cytokines. (E) Representative dot plots of CSF1R+ cells captured by FACS sorting or (F) magnetic beads. (G) Comparison of the monocyte distribution in the two sorting methods, plotting % of CSF1R+ cells (mean with SD, *n* = 3). (H) CSF1R expression in monocytes isolated with PBMCs with two different commercial isolation reagents, Ficoll or Optiprep (*n* = 3). (I) Comparison of CSF1R expression in fixed (*n* = 3) and non-fixed cells (*n* = 5). (J) CSF1R expression in freshly frozen cells (thawed), and in thawed cells immediately rested overnight in media at 37°C (rested) (*n* = 3). All data plotted mean with SD. One and two-way ANOVA tests, followed by Tukey and Sidak multiple comparison tests performed; *P < 0.05.

CSF1R expression has been demonstrated in monocytes of many species including birds, mice, cats, sheep, and pig [28]. In humans, studies by Ashmun *et al*. [11], Ingersoll *et al*. [13], and Loon-Wong [29] reported CSF1R expression in monocytes, however, its use in conventional human blood analysis was abandoned and mainly ascribed to CD16+ cells. Membrane CSF1R is stable at RT and at 4°C; however, it decreases in response to 2- and 4-hour stimulation with high CSF1 levels and even Toll-like receptor activation (Fig. 2A and B). Surface CSF1R continues to be decreased after 48 hours of cytokine and Toll-like receptor ligand exposure, but IL-4 and Dexamethasone provoke a slight increase over control. After 7 days of culture, monocytes mature into macrophages and CSF1R becomes more abundant than in monocytes in fresh blood. However, a range of hematopoietic cytokines did not alter CSF1R expression in this longer-term model (Fig. 2C and D).

We conclude that, as for other species, lower CSF1R levels in circulating monocytes can serve as proxy for increased levels of its ligands CSF1 and IL-34, or exposure to danger signals in the body. Our preferred CSF1R staining method which requires a simple 15-minute incubation of whole blood at RT has prognostic potential.

In the steady state, CSF1R membrane expression is sufficient to support selective isolation of all mononuclear phagocytes, via fluorescence sorting or with magnetic beads, versus all other white blood cells (Fig. 2E–G). Processing the blood with gradients of hydrophilic polysaccharide (Ficoll) or Iodixanol (Optiprep) does not affect detection provided thorough washing is done (Fig. 2H). Intensity differences between whole blood assays and peripheral blood mononuclear cell (PBMC) staining derive from altered antibody cell ratios in the staining. An important note is that fixation with paraformaldehyde or formaldehyde affects detection by this particular antibody clone (Fig. 2I). Staining of dimethyl sulfoxide and foetal bovine serum cryopreserved peripheral blood mononuclear cells is also possible, but cells need resting at 37°C to recover membrane levels of the receptor (Fig. 2J).

CSF1R as pan-phagocyte marker could revolutionise how we study monocyte and DC populations. To date, studying phagocytes requires multi-parametric analysis with a variety of receptors, e.g. CD14, CD16, CD1c, HLADR, CD11c, and exclusion of the so-called lineage markers CD3, CD19, CD20, CD56, among others. This results in loss of cell types and no other single marker is able to label all myeloid DCs and monocytes.

**Figure 3:**
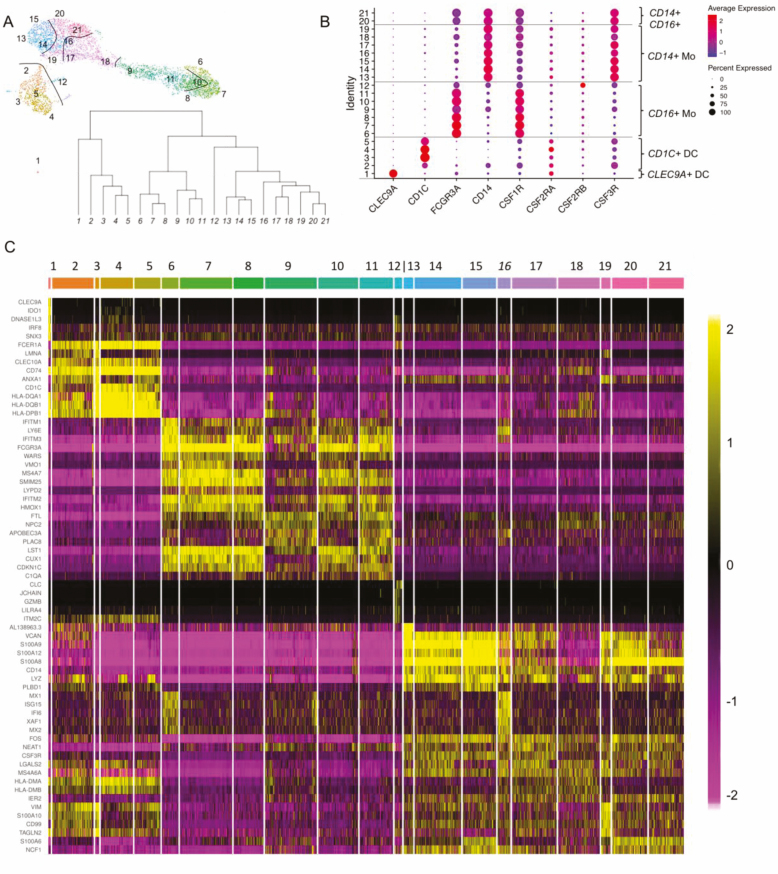
Single-cell RNAseq highlights the key CSF1R + members of the mononuclear phagocyte system in blood. (A) Single-cell RNA sequencing was performed on CSF1R+ cells (*n* = 3). The monocyte subset proportions were normalised before sequencing. UMAP clustering identified 21 unique clusters using FLOWSOM. The inset shows a hierarchical tree of cell clusters. (B) The expression of the LDCRs in each cluster was plotted in an abacus plot. Genes in each cell type can be observed in the heatmap, organised left to right using a hierarchical tree distance matrix. Four clear groupings of CD16+ and CD14+, CD14+CD16+ monocytes and DCs can be identified. (C) Heatmap of the top five differentially expressed genes in each of the 21 clusters, after redundancies were removed. Cluster 9 and 8 are double positive for CD14 and CD16 and are intermediate stages between CD14 and CD16. Cluster 12 and 16 which are DC-like according to the UMAP. Finally, Cluster 20 and 21, which appear positive for CD16, albeit dim.

To further characterise CSF1R as pan phagocyte marker, we performed single-cell (sc)RNAseq analysis of CSF1R+ cells from the blood of three healthy young donors. We normalised cell numbers collected in each CD14 CD16 quadrant by fluorescent sorting, to have equal representation of rare and abundant cells, such as DCs and CD14+CD16- monocytes, respectively. Uniform manifold approximation and projection (UMAP) analysis of the scRNAseq data shows that CSF1R+ cells fall under four main cell type transcriptomes, and at least 21 sub-transcriptome clusters (Fig. 3A).

The algorithm selects as most distant Cluster 1, which corresponds to the group of conventional cDC1 (CLEC9A+ CD141+ DCs) [30]. Cluster 2 are DC3 cells which express CD14 and CD1c. Cluster 3 and 4 are conventional cDC2, which express CD1c and lack CD14. Cluster 12 also appears close to DCs, suggestive of inflammatory iDCs [31]. In Cluster 13 to 19, we find CD14+ monocytes and Cluster 6 to 12 CD16+ monocytes. We are aware of DC specialisation [4], but monocyte diversity remains poorly understood. Double-positive monocytes are in fact heterogeneous, the abacus plot (Fig. 3B) reveals three double-positive alternatives: Cluster 9 and Cluster 18 are double-positive cells placed between single-positive cells, and Cluster 20 and 21 are also double positive but have a distinctive position closer to CD14+ cells (Fig. 3A). In Fig. 3A, we show the relationship between intermediate subsets with a hierarchical clustering tree, where again Cluster 20 and 21 are far from Cluster 9 and 18. Cluster 2 seems to contribute to Cluster 3, 4, 5. Within CD14+ monocytes, we have two extremes represented by single-positive cells in Cluster 13–15 and 6–8. There is a convergent CD16+CD14+ state in Cluster 9 to 18, which appear similar in UMAP, but are not hierarchically linked (Fig. 3A). In the heatmap (Fig. 3C), we can appreciate the transcriptome differences between the convergent states of Cluster 9 and 18; Cluster 2 and 12; and Cluster 20 and 21. We conclude that CSF1R is able to select all phagocytes of the monocyte and DC family in human, points to novel monocyte subsets, and excludes common confounders such as plasmacytoid DCs and polymorphonuclear cells.

COVID-19 has emerged as an atypical pathology, with a strong monocyte profile, with metabolic and trophic factor involvement, but lacking the inflammatory profile which makes other respiratory viruses lethal. The disease represents an exceptional example of a pathology with monocyte and macrophage dysregulation at its core. Lymphopaenia, neutrophilia, and monocyte dysregulation have also emerged as prototypic of COVID-19 [32, 33], but these are common to other inflammatory pathologies. Regarding monocytes, COVID-19 is associated with an increase in immature KI67+ cells, high CD169 Siglec-1 levels, and decreased HLADR; their activation is associated with blood vessel damage, coagulopathies, and organ failure, reviewed in ref. [34]. So far, studies focus on different severity levels of COVID-19, and it is not clear which mechanisms are unique to COVID-19 versus other lung pathologies.

We stained whole blood with a fluorescent multicolour FACS panel including CSF1R, CSF2R, CSF3R, IL3R, and CD14, CD16, and CD1c. White blood cell analysis confirms observations in COVID-19, with lymphopaenia and neutrophilia; however, lymphocytes also decreased in COVID-negative patients with chronic obstructive pulmonary disease (COPD), and in some asthmatic patients; similarly, neutrophils increased in all diseases evaluated, highlighting that these are general innate mechanisms not restricted to COVID-19 (Supplementary Fig. S3).

**Figure 4:**
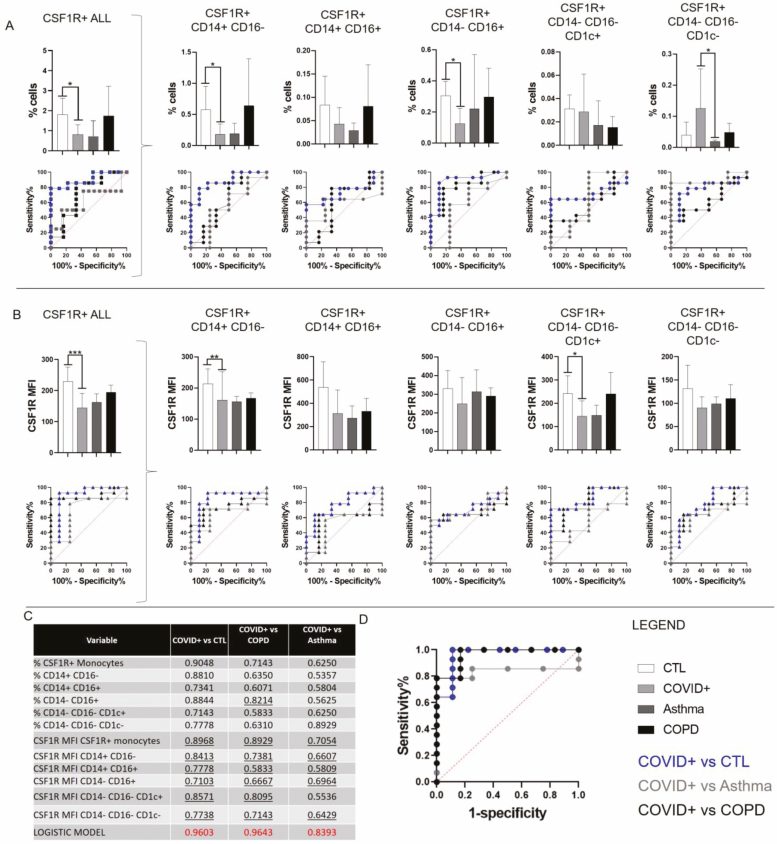
CSF1R measurement enhances COVID-19-specific ROC-AUC test scores. (A) We present the percentage of all CSF1R+ cells and divided according to CD14, CD16, and CD1c subsets. Samples were grouped into four categories, Healthy (*n* = 9), COVID+ (*n* = 14), Asthma (*n* = 4), and COVID- with COPD (*n* = 6). Bar charts represent means with SD. ROC-AUC curves were generated for each category versus COVID (Blue = COVID+ vs. CTL; Black = COVID+ vs. COPD; Grey = COVID+ vs. Asthma). Kruskal–Wallis and Dunn multiple comparison tests performed (*P < 0.05). (B) This panel is equivalent to panel A, but instead of the percentage of cells we report the expression levels of CSF1R. (C) ROC-AUC scores for every subset and comparison. Underlined values were used for multinomial logistic analysis. Scores can be categorised as 0.9–1 excellent, 0.8–0.9 good, 0.7–0.8 fair, 0.6–0.7 poor, and 0.50–0.6 fail. (D) With predictive logistic regression, we can combine parameters to create a uniquely powerful hyperparameter, which classifies COVID-19 from any of the conditions tested.

CSF1R is stably expressed in all healthy subjects, but in COVID-19, the CSF1R+ phagocyte numbers are reduced (Fig. 4A). This drop is shared with asthma, but not COPD. We investigated where the CSF1R decrease originates by sub-setting CSF1R+ cells into CD14+-CD16+- subsets and further divided the CD14-CD16- into CD1c+ and CD1c- populations. In COVID-19, there is a significant drop in CSF1R+ cells within the three conventional monocytes subsets. In contrast, CD14-CD16-CD1c+ cells remain unchanged, but there was a significant rise in CD14-CD16-CD1c- cells; these are so far undefined and could be expanding CD1c- DC subtypes and/or immature precursors. Asthma and COPD have different profiles lacking the CSF1R+CD14-CD16-CD1c- cells. We also examined CSF1R expression levels in all COVID-19 populations, and the changes are less pronounced, but equally significant (Fig. 4B). CSF1R MFI decrease is homogeneous and unimodal.

Receiver operator characteristic-area under the curve (ROC-AUC) analysis is valuable to determine discrimination ability of a marker. AUC indicates the probability that a randomly selected individual from the positive group has a positive test result, compared with a randomly selected individual from the control group. ROC-AUC curves for COVID versus healthy, asthma, or COPD, based on percentage of CSF1R+ cells or expression levels, show excellent discriminatory power (Fig. 4A and B and Supplementary Fig. S3). Importantly, with predictive logistic regression, we then put together selected scores, underlined in Fig. 4C, and created a model, where we can distinguish between all diseases with maximum confidence (Fig. 4D). The measures of association between predicted probabilities from the logistic model and observed responses such as Sommers’D, Gamma, and Tau-a are shown to be relatively strong for all models. Overall, the percentage of CSF1R+ cells or CSF1R membrane expression levels in all cells or specific subsets could distinguish COVID-19 from all our control groups.

## Conclusions

Monocytes and DCs originate predominantly in the bone marrow after birth [35, 36]. The cytokines M-CSF (CSF1) and IL-34 are the main ligands associated with their development and survival. CSF1R antibodies were initially described in the 80’s and identified in circulating monocytes. We use the cytokine to culture all monocytes and turn them into mature macrophages. Currently, the expression of CSF1R antigen remains unclear in human blood, and misconception about its expression in only selected monocyte subsets, arose from clinical trials [37]. Blood refrigeration and relatively fast processing is a requirement, similar to the staining of mouse monocytes [38]. Numerous CSF1R antibodies and small inhibitory molecules for the pathway have been developed by selected pharmaceutical companies but have been mostly abandoned due to partial benefits in a variety of diseases and clinical trials. We attribute the main reason for failure to the fact that monocytes and macrophages express more than only a single lineage cytokine receptor and they compensate each other when inhibited. Understanding the activity of anti-CSF1R drugs and pathophysiological mechanisms is hindered by poor understanding of lineage receptor expression.

CSF1R should change how we subset phagocytes in blood, how we study DC subsets, and finally provides a needed lineage marker for the family. We need to overcome the variability in strategies to sort and investigate the cells and bring innovation into this system. Bona fide markers of the MPS should not be shared with polymorphonuclear cells, nor should selecting the cells require complex multi-antibody strategies. The MPS systemic response is antigen non-specific, but incorporates innate and antigen-specific adaptive cell interactions. There is specificity in CSF1R regulation, and in COVID-19, CSF1R analysis can indicate the inflammatory status of patients and overcome limitations of virology, bacteriology, and antibody tests. CSF1R+ decrease could be due to higher CSF1 levels inducing internalisation of the receptor, and cell migration into tissues. A number of recent studies have indicated increased levels of CSF1 in the serum of COVID+ patients [39–41]. Deng *et al*. specially highlighted a correlation in the levels of CSF1 to the inflammatory cytokine IL-6, and furthermore demonstrated CSF1 ability to discriminate between healthy and COVID+ patients.

We have shown that changes in CSF1R expression in specific subsets can segregate COVID-19 patients with confidence. Blood is readily accessible to detect MPS changes in COVID-19, unlike bone marrow and tissue biopsies and post-mortem specimens. In clinical terms, it means that in less than 1 hour, we can classify patients according to pathology, the contribution of the biomarker to prognosis, and determine which patient subgroup needs investigation. The biggest limitation of our study is sample numbers, age matching, and severity studies, which could be achieved at a global level.

The mononuclear phagocyte lineage as determined with the CSF1R, provides approaches to better understand mechanisms of inflammation and MPS activation, pointing to drug and cellular targets to prevent and treat diverse infections beyond COVID-19, as well as immune and autoimmune host responses, malignancy, and degenerative diseases. Inhibitory monotherapy for CSF1R, or combination therapy with anti-CSF2R, has the potential to replace the widely used, yet deleterious steroid treatment. Rediscovery of the CSF1R as a reproducible lineage biomarker of mononuclear phagocytes in human blood opens the door to integrated studies of innate and adaptive immunity in health and disease, patient stratification in the clinic, and future anti-inflammatory therapies for COVID-19 and other infections or malignant diseases.

## Supplementary material

Supplementary data are available at *Immunotherapy Advances* online.

Figure S1. Flow cytometry controls for the panel of lineage determining cytokine receptors.

Figure S2. CSF2R selects for CD14+ monocytes.

Figure S3. CSF1R measurement enhances COVID-19- specific ROC-AUC test scores.

Figure S4. Flow cytometry sorting gating strategy.

Table S1. Demographics of the COVID-19 cohort. T2DM – Type 2 Diabetes Mellitus; HTN –Hypertension; COPD – Chronic obstructive pulmonary disease.

Table S2. MFI values for unstained background (n=5), FMO controls (n=2) and antibody staining (n=5) for each receptor and for each leukocyte.

ltab003_suppl_Supplementary_Materials

## Figures and Tables

**Table:** 

Melanie Bailey	Natalie Riddell	Alex Stewart
Emma Sinclair	Phil Morgan	Manjula Meda
Nora Kasar	Gill Wallace	Matt Spick
Katie Longman	Vasiliki Tsioligka	Thanuja Weerasinghe
Cecile Frampas	Fernando Martinez Estrada	Joanne Zamani
Catia Costa	Deborah Dunn-Walters	Federica Orsenigo

## Abbreviations

COPD: Chronic obstructive pulmonary disease
CSF1R: Colony stimulating factor 1 receptor
CSF2R: Colony stimulating factor 2 receptor
CSF3R: Colony stimulating factor 3 receptor
DC: Dendritic cell
FACS: Fluorescent activated cell sorting
FLT3: fms-like tyrosine kinase 3
IL3R: Interleukin 3 receptor
IL-7R: Interleukin 7 receptor
IL-15R: Interleukin 15 receptor
LDCRs: Lineage determining cytokine receptors
MFI: Median fluorescent intensity
MPS: Mononuclear phagocyte system
PBMC: Peripheral blood mononuclear cell
ROC-AUC: Receiver operator characteristic- area under the curve
UMAP: Uniform manifold approximation and projection.

## Data and availability

There are no restrictions on data availability.
