# Supplementary material for: CSF1R defines the mononuclear phagocyte system lineage in human blood in health and COVID-19
Source: Immunother Adv. 2021 Feb 17;1(1):ltab003. doi: 10.1093/immadv/ltab003 (PMC7928847; doi:10.1093/immadv/ltab003)
Supplement: ltab003_suppl_Supplementary_Materials [file ltab003_suppl_supplementary_materials.docx]

Supplementary Materials for

Title: CSF1R defines the Mononuclear Phagocyte System lineage in human blood in health and COVID-19

**Authors:** Theo Combes, Federica Orsenigo, Alex Stewart, A. S. Jeewaka R. Mendis, Deborah Dunn-Walters, Siamon Gordon, Fernando O. Martinez

**This file includes:**

Figures S1 to S4

Captions for Figures S1 to S4

Table S1, S2

**Supplementary Figures**

**Figure S1. Flow cytometry controls for the panel of lineage determining cytokine receptors. A)** Representative dot plots and histograms for single antibody staining performed in whole blood. Abbreviations: SSC-A–Side scatter area, Mono–Monocytes; PMN–Polymorphonuclear cells; Lymph–Lymphocytes. **B)** For our study we used a multicolor panel containing live and dead staining, CD14, CD16 and the 7 LDCR. Here we show the expression of each of LDCR in the full multicolor panel and with the fluorescent minus one (FMO) control.

**Figure S2. CSF2R selects for CD14+ monocytes. A)** representative dot plot showing CSF2R expression in whole blood leukocytes. **B)** CSF2R+ cells were selected and separated according to CD14 and CD16 expression, showing it selects for CD14+ monocytes. **C)** Distribution of the monocyte subsets from CSF1R or CSF2R selection. CSF1R captures significantly more CD14-CD16+. We plotted % means SD of cells in each CD14-|CD16 quadrant, with % of CSF1R+ versus CSF2R+ cells (n=5). Two-way ANOVA and Sidak’s multiple comparison test performed, ****=p<0.0001.

**Figure S3. CSF1R measurement enhances COVID-19- specific ROC-AUC test scores.** A) The percentage of each leukocyte in the blood was identified. Lymphocytes and neutrophils were delineated based on SSC-A and FSC-A as in Figure 1. Monocytes were selected based on CSF1R or CD14 positivity. B) ROC curves were generated from the above cell percentage data, comparing COVID+ patients (n=14) vs CTL (n=9) (Blue), COPD (n=6) (Black) and Asthma (n=4) (Grey) samples. All data plotted mean with SD. Kruskal-Wallis and Dunn multiple comparison tests performed (*=p<0.05).

**Table S1.** Demographics of the COVID-19 cohort. T2DM – Type 2 Diabetes Mellitus; HTN – Hypertension; COPD – Chronic obstructive pulmonary disease.

**Table S2.** MFI values for unstained background (n=5), FMO controls (n=2) and antibody staining (n=5) for each receptor and for each leukocyte.

**
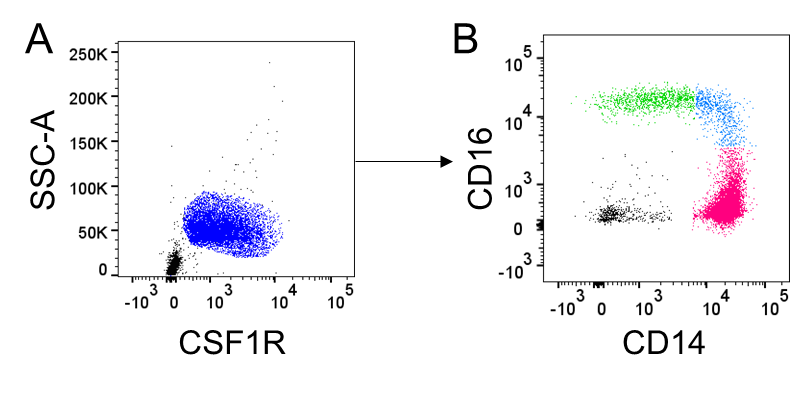
**

**Figure S4. Flow cytometry sorting gating strategy.** **A)** CSF1R+ve cells (in blue) in the PBMCs are gated, leaving the lymphocytes in black. **B)** The CSF1R+ cells are then subdivided into four populations based on CD14 and CD16 expression. The four populations are sorted.
